# The ECM Modulator ITIH5 Affects Cell Adhesion, Motility and Chemotherapeutic Response of Basal/Squamous-Like (BASQ) Bladder Cancer Cells

**DOI:** 10.3390/cells10051038

**Published:** 2021-04-28

**Authors:** Michael Rose, Erik Noetzel, Jennifer Kistermann, Julian Eschenbruch, Sandra Rushrush, Lin Gan, Ruth Knüchel, Nadine T. Gaisa, Edgar Dahl

**Affiliations:** 1 Institute of Pathology, University Hospital RWTH Aachen University, 52074 Aachen, Germany; jenny.kistermann@googlemail.com (J.K.); sandrarushrush@hotmail.com (S.R.); rknuechel-clarke@ukaachen.de (R.K.); ngaisa@ukaachen.de (N.T.G.); 2 Institute of Biological Information Processing 2 (IBI-2), Mechanobiology, Forschungszentrum Jülich GmbH, 52425 Jülich, Germany; e.noetzel-reiss@fz-juelich.de (E.N.); j.eschenbruch@fz-juelich.de (J.E.); 3 IZKF Aachen, Medical Faculty, RWTH Aachen University, 52074 Aachen, Germany; lgan@ukaachen.de

**Keywords:** bladder cancer, BASQ subtype, ITIH5, hyaluronan, CD44, chemosensitizer

## Abstract

This study aims at characterizing the role of the putative tumor suppressor ITIH5 in basal-type bladder cancers (BLCA). By sub-classifying TCGA BLCA data, we revealed predominant loss of ITIH5 expression in the basal/squamous-like (BASQ) subtype. ITIH5 expression inversely correlated with basal-type makers such as KRT6A and CD44. Interestingly, Kaplan–Meier analyses showed longer recurrence-free survival in combination with strong CD44 expression, which is thought to mediate ITIH-hyaluronan (HA) binding functions. In vitro, stable ITIH5 overexpression in two basal-type BLCA cell lines showing differential CD44 expression levels, i.e., with (SCaBER) and without squamous features (HT1376), demonstrated clear inhibition of cell and colony growth of BASQ-type SCaBER cells. ITIH5 further enhanced HA-associated cell-matrix attachment, indicated by altered size and number of focal adhesion sites resulting in reduced cell migration capacities. Transcriptomic analyses revealed enrichment of pathways and processes involved in ECM organization, differentiation and cell signaling. Finally, we provide evidence that ITIH5 increase sensitivity of SCaBER cells to chemotherapeutical agents (cisplatin and gemcitabine), whereas responsiveness of HT1376 cells was not affected by ITIH5 expression. Thus, we gain further insights into the putative role of ITIH5 as tumor suppressor highlighting an impact on drug response potentially via the HA-CD44 axis in BASQ-type BLCA.

## 1. Introduction

Inter-α-trypsin inhibitory heavy chain 5 (ITIH5) belongs to the ITI gene family [1] comprising additional heavy chains (ITIH11–3 and 6) involved in stabilization of the extracellular matrix (ECM) [2]. Analogously to heavy chain 1–3, ITIH5 is thought to covalently bind hyaluronan (HA) via a conserved cleavage site, unmasking the C-terminal amino acid interacting with HA [3,4,5]. HA, a major component of the ECM [6], affects a spectrum of biological activities including cell motility, wound healing and differentiation mediated by HA binding proteins (HABP), in particular, membrane- bound receptors such as CD44 and RHAMM [7,8,9]. Crystal structures of CD44 complexed with HA showed different conformational transition forms of CD44 due to HA binding which is thought to modulate downstream signaling [10]. So far, different CD44 splice variants have been described to affect tumorigenesis, cancer stemness and drug resistance [11,12,13], e.g., CD44v3 promotes progression of urothelial carcinomas through AKT/ERK/STAT3 pathways [14]. In 2016 Martin et al. described an HA-dependent control of the fibroblast phenotype by ITIH5 [15], i.e., ITIH5 stabilization of HA was essential for a TGFβ1-driven differentiation of fibroblasts to myofibroblasts initiated by HA-dependent co-localization of CD44 and epidermal growth factor receptor (EGFR). Recently, Huth and colleagues confirmed stabilization of HA-cable-like structures by ITIH5 in the skin [16], while ITIH5 dysregulation was associated with diverse pathological processes including inflammatory diseases such as allergic contact dermatitis [17].

Since 2004, ITIH5 has been reported to block tumor growth, migration and metastasis of various types including breast [18], pancreatic [19] and bladder cancer [20]. In breast cancer, ITIH5 modulates the TGF-β signaling cascade via the co-receptor endoglin which impairs metastases promoters’ like ID1 [21]. Moreover, ITIH5 was causative for an epigenomic reprogramming of basal-type breast cancer cells upon a shift from mesenchymal to an epithelial-like phenotype while suppressing lung metastases formation in vivo [22]. ITIH5 overexpression further altered focal adhesions and cell contractility-based forces involving mechanotransductive integrin-GTPase signaling [22]. In bladder carcinogenesis, we previously demonstrated epigenetic inactivation of ITIH5 associated with advanced tumor stages predicting unfavorable prognosis of patients diagnosed with a papillary (pT1) urothelial high-grade tumor [20]. ITIH5 promoter methylation was also characterized as putative biomarker for non-invasive detection of breast [23] and bladder cancer [24] via plasma and urine samples, respectively.

Still, bladder cancer is the most frequent urogenital malignant tumor concerning both sexes worldwide with estimated ~549,400 new cases and 200,000 deaths [25]. Histologically, bladder cancer comprises a heterogeneous spectrum including urothelial cancers with squamous differentiation, which have been associated with poor response to chemotherapy [26,27]. Since urothelial cancers have been further classified into distinct molecular subtypes [28,29] confirming a basal-type with squamous features (BASQ) associated with poor prognosis [30], we aimed to give additional insights into ITIH5 expression and its functional and therapeutic implications for this clinically important subgroup.

## 2. Results

### 2.1. ITIH5 Loss Predominates in BASQ Bladder Cancers, While the Prognostic Impact Is Associated with Hyaluronic Receptor Expression

In 2014, the prognostic impact of the tumor suppressor ITIH5 has been studied in bladder cancer [20], however, its function in basal-type bladder cancer remained poorly understood. Since we demonstrated a significant role of ITIH5 on basal-type breast cancer cells [22], we here assessed *ITIH5* mRNA expression according to the sub-classification of high-grade bladder that has previously been shown to reflect hallmarks of breast cancer biology [31].

**Figure 1:**
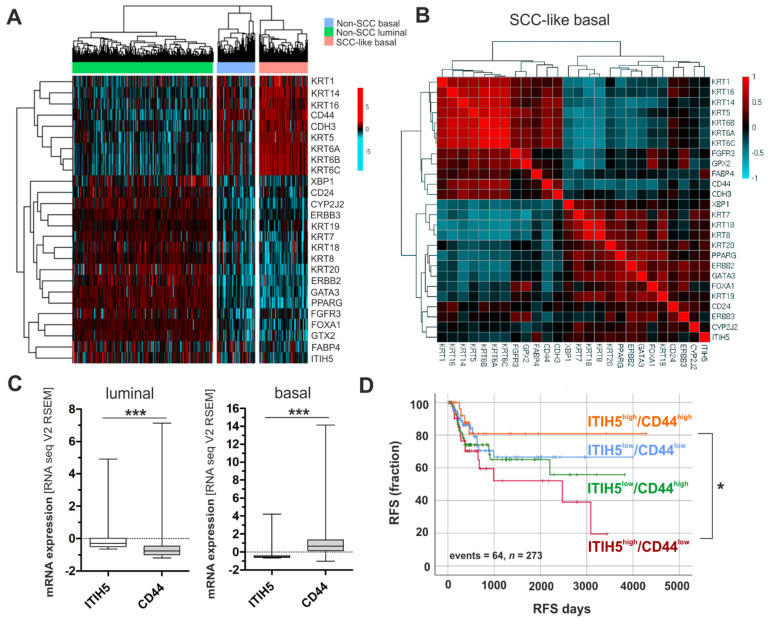
ITIH5 is predominantly down-regulated in basal-type bladder cancers, while maintained ITIH5 expression predicts longer recurrence-free survival in association with the basal marker CD44. (**A**) Heatmap cluster analysis of basal-type and luminal-type markers in distinct bladder cancer subtypes of the TCGA data set reveals low ITIH5 mRNA in association with basal-type bladder cancers. (**B**) Non-parametric Spearman’s analysis to illustrate correlations between mRNA expression of basal-type, luminal-type markers and ITIH5 in the subgroup of SCC-like bladder cancers visualized by a pairwise matrix; red: strong positive correlation; blue: strong negative correlation. (**C**) Box plots show inverse *ITIH5* and *CD44* mRNA expression if comparing luminal and basal-type bladder cancers: correlation coefficient including all bladder tumors of the TCGA data set: r: −0.182, *p* < 0.001. (**D**) Kaplan–Meier survival curves illustrating recurrence-free survival (RFS) of patients stratified by different combinations of *ITIH5* and *CD44* mRNA expression: ITIH5^low^/CD44^low^ (blue curve), ITIH5^high^/CD44^low^ (red curve), ITIH5^low^/CD44^high^ (green curve), ITIH5^high^/CD44^high^ (orange curve). Vertical lines: censored cases. * *p* < 0.05; *** *p* < 0.001.

The Cancer Genome Atlas (TCGA) bladder cancer data (*n* = 386) were classified into distinct molecular subtypes, i.e., luminal, basal and basal-squamous (BASQ) by applying gene signatures as previously specified [32]. A cluster analysis of luminal and basal-type markers including ITIH5 expression revealed differences among intrinsic bladder cancer subtypes: Basal-type bladder cancers were characterized by abundant ITIH5 mRNA expression loss (Figure 1A). Interestingly, in the basal-type subgroup of bladder cancers with SCC features (BASQ), an inversely ITIH5 expression with basal-type markers such as KRT6A has been confirmed, whereas a strong positive correlation of remained (low) *ITIH5* expression with (low) expression of luminal-type markers like KRT20 was present (Figure 1B), i.e., in tumors where substantially basal-type markers are predominant, a close association between *ITIH5* and luminal-type markers could be still observed on mRNA level. A negative correlation was also shown between expression of ITIH5 and the basal-type marker CD44 (Figure 1B,C), which is thought to mediate ITIH-HA binding functions as described before. Interestingly, ITIH5 expression predicted longer recurrence-free survival only in combination with strong CD44 expression independently of a given subtype (Figure 1D). In contrast ITIH5 expression was associated with poor patients’ outcome in case of lacking CD44 expression.

**Figure 2:**
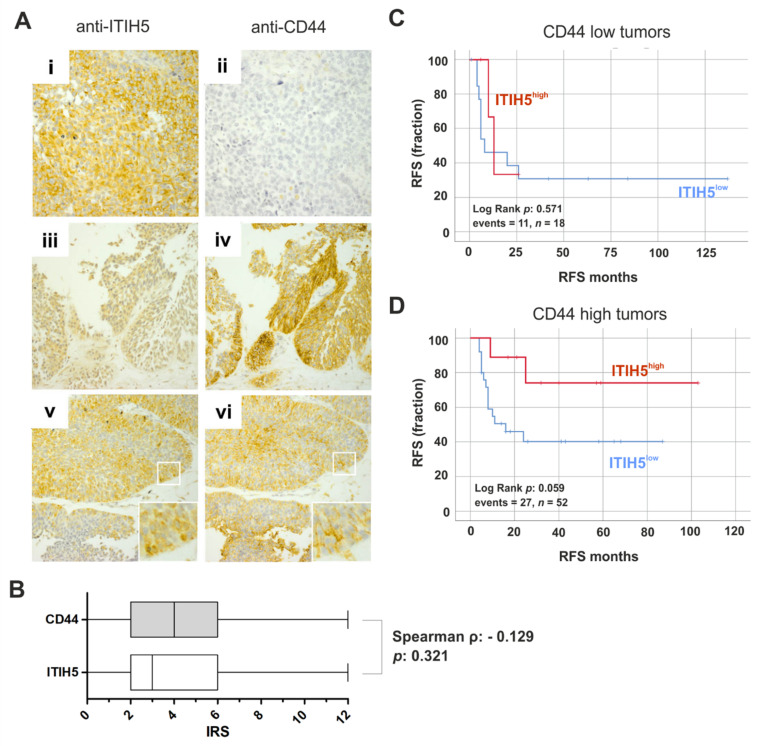
ITIH5 and CD44 protein expression in bladder cancer cells. (**A**) Immunohistochemical staining of ITIH5 and CD44 protein of representative bladder cancers are shown. (i–ii) Strong ITIH5 but no CD44 immunoreactivity; (iii–iv) Low ITIH5 and strong CD44 immunoreactivity; (v–vi) moderate co-expression of ITIH5 and CD44. Original magnification: 400× (i + ii) and 100× (iii–vi). (**B**) Box plot illustrates ITIH5 and CD44 protein expression (IRS) in n = 52 bladder cancer tissues. A Spearman’s correlation coefficient calculation showed tendency of an inverse expression, but significance failed. (**C**,**D**) Kaplan–Meier survival curves display RFS of patients with low ITIH5 protein expression (blue curve; immunoreactive score (IRS) 0–6) compared to strong ITIH5 protein expression (red curve; IRS 8–12) in bladder cancer with low (**C**) and high (**D**) CD44 expression.

Next, we stained n = 52 bladder cancer tissues for ITIH5 and CD44 protein expression by immunohistochemistry. Representative images of stained tissue slides of tumors with inverse and co-expressed ITIH5-CD44 protein are shown in Figure 2A. Potentially due to the limited number of samples, an inverse correlation could not be statistically confirmed (Figure 2B). Stratifying the tumor samples by CD44 expression into low and high expressed groups, Kaplan–Meier analyses revealed that ITIH5 expression tend (*p* = 0.059) to predict a longer RFS (ΔRFS: 40.6 months) in bladder cancer with strong CD44 expression (Figure 2C), while its expression completely missed significance in CD44-low expressed tumors (Figure 2D).

### 2.2. ITIH5 Re-Expression Inhibits Cell and Colony Growth of BASQ Bladder Cancer Cells In Vitro

**Figure 3:**
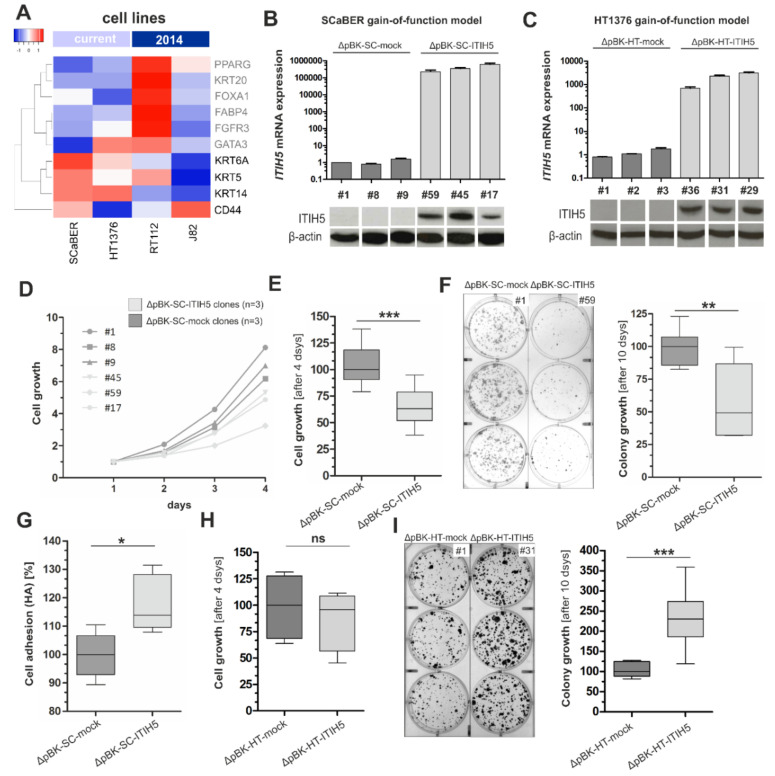
Basal-type bladder cancer cell lines overexpressing ITIH5: Tumor and colony growth are impaired whereas cell adhesion on HA is facilitated by ITIH5 overexpression in CD44^high^ SCaBER cells. (**A**) mRNA expression (TCGA data set) of selected luminal (gray) and basal-type (black) markers including CD44 in bladder cancer cell lines used in this (SCaBER and HT1376) and our previous ITIH5 study (RT112 and J82, see [20]). SCaBER and HT1376 cells express basal-type markers in a comparable manner except for CD44: While SCaBER cells show abundant CD44 expression (denoted CD44^high^), HT1376 cells exhibit low CD44 (denoted CD44^low^). Please note: Expression level does not reflect absolute expression values—only a relative expression value is given by array-based data—and variants of CD44 are also not considered. (**B**,**C**) ITIH5 gain-of-function models of basal bladder cancer lines based on (**B**) SCaBER and (**C**) HT1376: Specific ITIH5 mRNA overexpression in stable SCaBER (ΔpBK-SC-ITIH5) and HT1376 (ΔpBK-HT-ITIH5) single-cell clones were further confirmed by Western blotting compared to mock control clones. β-actin served as loading control. (**D**,**E**) XTT proliferation assay was performed. SCaBER ITIH5 single-cell clones showed reduced cell growth compared with ΔpBK-SC-mock controls. The baseline level optical density (OD) at 24 h was set to 1. (**F**) Long-term colony growth of basal SCaBER bladder cancer cells upon ITIH5 expression. Box plot presents averages of triplicate experiments based on three independent ScaBER ΔpBK-SC-ITIH5 and three ΔpBK-SC-mock clones. Left: Representative wells with grown ΔpBK-SC-ITIH5 as well as ΔpBK-SC-mock colonies are shown. Right: Densitometrical evaluation of colony growth after 10 days. (**G**) Cell-to-matrix adhesion of ΔpBK-SC-ITIH5 and ΔpBK-SC-mock clones on HA-substrate. (**H**) Short-term XTT proliferation assay was performed. HT1376 single-cell clones overexpressing ITIH5 did not show altered cell growth compared with ΔpBK-HT-mock controls after 96 h. (**I**) Long-term colony growth of basal HT1376 bladder cancer cells upon ITIH5 expression. Box plot presents averages of triplicate experiments based on three independent HT1376 ΔpBK-HT-ITIH5 and three ΔpBK-HT-mock controls. Left: Representative wells with grown ΔpBK-HT-ITIH5 as well as ΔpBK-HT-mock colonies are shown. Right: Densitometrical evaluation of colony growth after 10 days. ns: not significant, * *p* ≤ 0.05, ** *p* < 0.01, *** *p* < 0.001.

Facing the expression loss of ITIH5 in basal-type bladder cancers, we further aimed to analyze the functional impact of this tumor suppressor gene on basal-type bladder cancer cells in vitro. We selected cell line models for basal-type bladder cancers with squamous features (SCaBER cell line [33]) and without (HT1376), in addition showing differential CD44 expression levels (Figure 3A). Stable single-cell clones were generated using a full-length ITIH5 cDNA pBK-CMV expression vector (pBK-ITIH5 clones) or empty vector (pBK-mock clones) based on the basal-type, SCC-like bladder cancer cell line SCaBER (SC clones) and HT1376 (HT clones) (Figure 3B,C).

In monoclonal SCaBER cells, forced ITIH5 expression inhibits the median cell growth by 37.04% over 96 h compared to mock transfected clones (Figure 3D,E). Colony growth was studied over 10 days and macroscopic assessment of grown colonies visualized impaired colonization mediated by ITIH5 overexpression in SCaBER cells (Figure 3F). Densitometric evaluation of grown colonies significantly confirmed blocked colony growth in ITIH5-expressing SCaBER clones (n = 3) by 50.63% compared to independent ΔpBK-mock control clones. In parallel, ITIH5 altered cell-matrix adhesion of SCaBER cells: On hyaluronan-coated cell dishes, cell-matrix adhesion was significantly increased (13.80%, *p* < 0.001) in ΔpBK-ITIH5 SCaBER clones compared to mock control clones (Figure 3G). Contrary to that, basal-type HT1376 bladder cancer cells overexpressing ITIH5 did not alter short-term cell growth (Figure 3H). Interestingly, colony growth was significantly fostered over 10 days in ΔpBK-ITIH5 HT1376 clones (130.7%, *p* < 0.01) compared to mock control clones (Figure 2I).

### 2.3. ITIH5 Re-Expression Impairs Cancer Cell Migration Associated with Alterations in Cell-Matrix Adhesion of BASQ Bladder Cancer Cells In Vitro

Next, we aimed to assess the involvement of cancer progression-related biological processes and pathways in basal-type bladder cancer cells with squamous features potentially affected by the ITIH5-HA-CD44 axis. Transcriptomic analyses were performed of three independent ITIH5 overexpressing SCaBER clones and three independent mock clones. Applying gene set enrichment analyses (gene ontology (GO)), we revealed ITIH5 associated up- or down-regulation of genes involved in extracellular matrix organization (Table 1) which is in line with previous reports and findings in basal-type breast cancer cells [22].

In addition, *n* = 140 co-regulated and *n* = 159 anti-regulated genes were identified which met the following criteria: Significantly (raw *p* < 0.01) differentially expressed between ITIH5 and mock clones with a minimal change in expression by 1.25-fold. Significantly up- and down-regulated genes are summarized in Supplementary Table S1. Gene set enrichment analyses via the Molecular Signature Data Base (MSigDB) [34] revealed overlap of the pattern of *n* = 299 significantly de-regulated genes with signatures associated, for instance, with regulation of cell differentiation (genes in overlap: 38; *p* = 3.33 × 10 − 8), signaling receptor binding (genes in overlap: 35: *p* = 1.76 × 10 − 8), or collagen containing extracellular matrix (genes in overlap: 16; *p* = 1.21 × 10 − 7) (see Supplementary Table S2).

**Figure 4:**
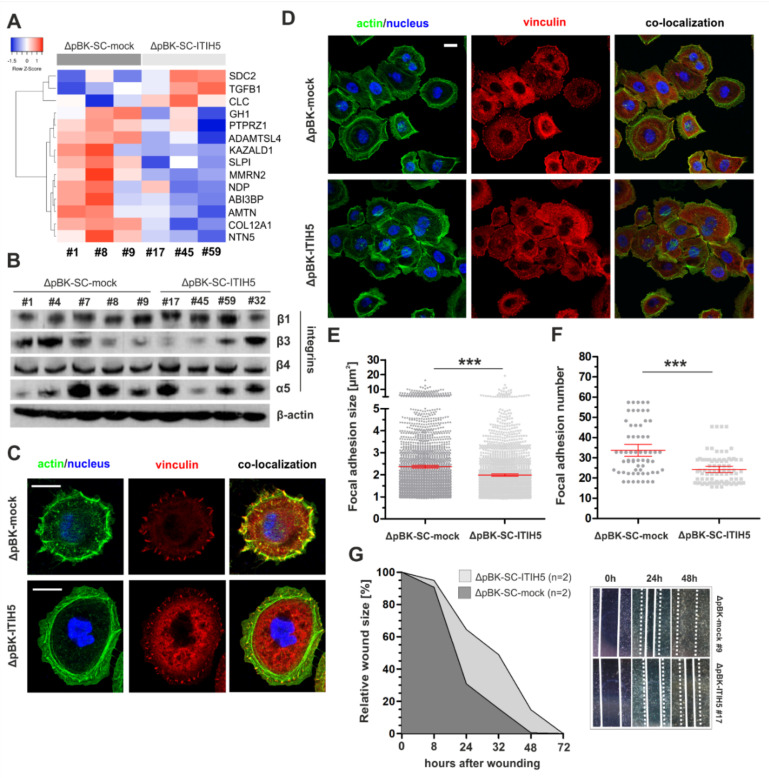
ITIH5 expression modulates cell-ECM interactions associated with impaired motility of SCaBER cells. (**A**) Heatmap illustrates array-based gene expression analysis of three ΔpBK-SC-ITIH5 and three ΔpBK-SC-mock clones. Presented genes are significantly altered due to ITIH5 expression in SCaBER cells and are associated with the GO:0062023: collagen-containing extracellular matrix. (**B**) Western blot illustrates unaltered protein levels of focal adhesion-related integrins in ΔpBK-SC-ITIH5 and ΔpBK-SC-mock clones. β-actin served as loading control. (**C**) Representative actin (green), vinculin staining (red) and merged (green/red; co-localization) of single ΔpBK-SC-mock and ΔpBK-SC-ITIH5 cells highlight the changed organization of the F-actin cytoskeleton and FA formation on fibronectin (FN) after ITIH5 overexpression. Scale bar: 20 µm. (**D**) Representative confocal images illustrate the cell cluster morphology of ΔpBK-SC-ITIH5 and ΔpBK-SC-mock clones adhered on FN-functionalized glass substrates. The actin cytoskeleton (green) with prominent stress fiber formation and the mechanosensor protein vinculin (red) are shown. Co-localization of vinculin patches in stress fibers indicates cell force transmitting focal adhesions. Scale bar: 20 µm. (**E**,**F**) Quantification of FA site formation. Overall n = 67 cells (FA number = 2260) of mock clones and n = 85 cells (FA number = 2057) of ITIH5-expressing clones were analyzed. Vinculin patches served as marker indicating the number and maturation state (size) of FA sites. FA number were normalized against the cell number. ΔpBK-SC-ITIH5 clones are characterized by significant reduction of FA size (**E**) and FA number (**F**) when compared to ΔpBK-SC-mock clones. (**G**) Cell migration was analyzed by using a wound healing assay. Mean rate of wound closure by ΔpBK-SC-ITIH5 and ΔpBK-SC-mock clones (n = 2) was analyzed over 72 h. Note that ITIH5 clones close the wound significantly slower. Cell-free area on day 0 was set as 100% and used for standardization. *** *p* < 0.001.

A set of significantly enriched genes as part of the GO term: “collagen containing extracellular matrix” is shown as a heatmap in Figure 4A. According to an overview of the MSigDB gene sets by categorizing genes into a number of carefully chosen “gene families”, the ITIH5-associated gene pattern includes *n* = 13 cytokines/growth factors (e.g., TGFβ1), *n* = 16 transcription factors (e.g., HOXA13), n = 11 cell differentiation markers (e.g., TLR3) *n* = 7 kinases (e.g., WEE2), *n* = 4 oncogenes (e.g., CDX2) and *n* = 2 tumor suppressor genes (e.g., CBLB).

Given the close similarity of these findings with previous data sets found in basal-type breast cancer [22], we analogously analyzed focal adhesion sites and associated factors involved in cell-matrix interaction. Western blot analyses showed heterogeneous but unchanged protein expression levels of FA integrins (Figure 4B). The α5β1 integrins known to mediate binding to fibronectin (FN) [35] were most similar expressed between clones, thus suitable to assess FA formation in dependency of ITIH5 expression on fibronectin-coated grounds (Figure 4C,D). The subcellular localization of the FA marker vinculin was distinct in ITIH5 clones (ΔpBK-SC-ITIH5 #59) compared to ΔpBK-SC-mock clone #9. A quantitative evaluation verified a significant reduction of FA formation size in ITIH5-expressing clones (mean FA size: 1.99 µm^2^; 95% CI: 1.94–2.04) compared to mock clones (mean FA size: 2.37 µm^2^; 95% CI: 2.31–2.43)) (Figure 4E). Significant changes in stress fibers were not confirmed. In turn, ITIH5-expressing cells showed a clear reduction of the mechanosensory protein vinculin reflecting decreased number of FA per cell of ITIH5-expressing clones (mean FA number: 24.2; 95% CI: 22.7–25.7) compared to controls (mean FA number: 33.7; 95% CI: 30.8–36.7) (Figure 4F). This could implicate for decreased FA-mediated cell-contractility of ITIH5-expressing BASQ cells compared with those lacking ITIH5. Such a decreased FA formation and maturation was present in ΔpBK-SC-ITIH5 cell cluster as well as in single migrating cells. ITIH5 re-expression in BASQ cells was associated with a characteristic reorganization of contractile cortical actin stress fibers and cell-matrix adhesion sites. Thus, we next investigated the impact of the observed changes in matrix adhesion on related cell function, i.e., cell migration and cluster formation. In parallel, studying cell migration by performing a wound healing assay, we showed a reduced cell migration capability of basal-type SCaBER cells upon forced ITIH5 expression, i.e., SCaBER ΔpBK-mock clones repopulated the wounded area notably faster than corresponding ITIH5-expressing single-cell clones over three days (72 h). The mean rate of cell repopulation within the scratched area by ITIH5 overexpressing and mock control clones is shown in Figure 4G. Already 24 h after cell wounding, ΔpBK-SC-mock clones repopulated 30.86% of the wound, whereas the SCaBER ΔpBK-SC-ITIH5 clones covered on average 64.55% (Figure 4G).

### 2.4. ITIH5 Re-Expression Increases Sensitivity of BASQ Bladder Cancer Cells upon Chemotherapeutic Treatment

Since ECM [36] and CD44 associated mechanisms [13] have been shown to be involved in chemotherapeutic response, we finally performed functional drug-response analyses using SCaBER and HT1376 cells to calculate relative IC_50_ values (half maximal inhibitory concentration) for mock and ITIH5-transfected single-cell clones upon cisplatin and/or gemcitabine treatment.

**Figure 5:**
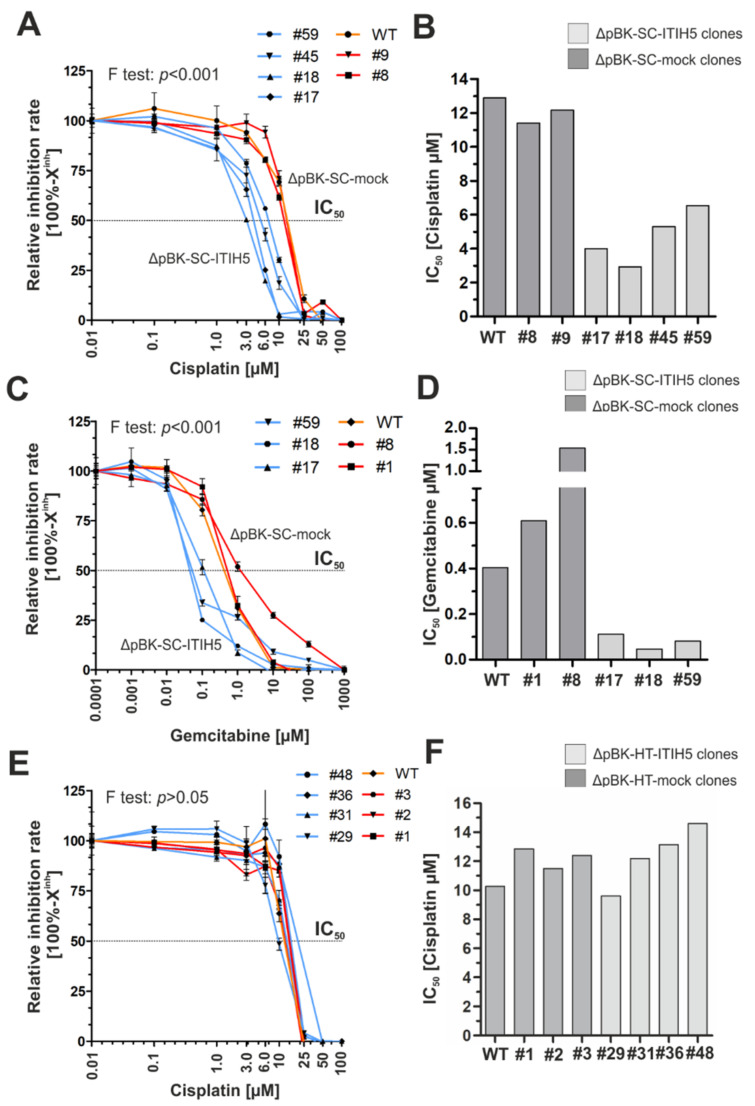
ITIH5 expression sensitizes SCaBER cells to cisplatin and gemcitabine treatment in vitro. (**A**–**C**) Semi-logarithmic plots show drug response curves (relative inhibition rate = 100% − X^inh^) for cisplatin and gemcitabine are shown. Drug response was determined using XTT following 72 h incubation with indicated drug concentrations. (**A**) Drug response of SCaBER WT (orange line), ΔpBK-SC-ITIH5 (blue lines) and ΔpBK-SC-mock (red lines) clones upon cisplatin treatment. (**B**) Drug response of SCaBER WT (orange line), ΔpBK-SC-ITIH5 (blue lines) and ΔpBK-SC-mock (red lines) clones upon gemcitabine treatment. (**C**) Drug response of HT1376 WT (orange line), ΔpBK-HT-ITIH5 (blue lines) and ΔpBK-HT-mock (red lines) clones upon cisplatin treatment. (**D**–**F**) Relative IC_50_ (drug concentration causing 50% inhibition) values are derived from the drug response curve to define the sensitivity of each single-cell clone and WT, respectively.

Following the extra sum-of-squares F test, drug response curves are distinct and have substantially different IC_50_ values in SCaBER clones, i.e., the null hypothesis (IC_50_ same for all data set) was significantly (*p* < 0.001) rejected. SCaBER clones overexpressing ITIH5 were, by far, much more sensitive compared to mock control clones upon treatment with both cytostatic drugs, i.e., cisplatin (ΔpBK-SC-ITIH5: mean IC_50_ = 4.69 µM; ΔpBK-SC-mock: 11.78 µM) and gemcitabine (ITIH5 clones: mean IC_50_ = 0.08 µM; mock clones: 1.07 µM) (Figure 5A–D). Responsiveness of mock clones was very comparable to the IC_50_ value of the WT SCaBER cells treated with cisplatin (IC_50_ = 12.89 µM) and gemcitabine (IC_50_ = 0.41 µM). In HT1376 cells, ITIH5 expressing did not significantly modulate responsiveness due to chemotherapeutic application (F test > 0.05; i.e., IC_50_ same for all data sets). Upon cisplatin treatment, the mean IC_50_ of ITIH5 overexpressing and correspondent mock clones was not altered (ΔpBK-HT-ITIH5: mean IC_50_ = 12.38 µM; ΔpBK-HT-mock: 12.25 µM) (Figure 5E,F).

## 3. Discussion

The tumor suppressor protein ITIH5 has been reported to be down-regulated in many cancer entities, e.g., breast cancer [18], colon cancer [37], lung cancer [38], cervical cancer [39], gastric cancer [40], pancreatic cancer [19] and bladder cancer [20]. In the presented study, we observed abundant loss of ITIH5 expression in the basal-type subgroup of bladder cancer similar to our findings in breast cancer [22]. In turn, ITIH5 expression was tightly associated with luminal-type markers such as KRT20. This observed association was still present in the BASQ subgroup suggesting a tumor suppressive role in dependency of cellular differentiation or even directly affecting differentiation analogously to basal-type breast cancer cells [22]. Interestingly, we did not observe any prognostic impact of ITIH5 expression alone in the BASQ subgroup. However, considering CD44, a basal-type marker but also a putative mediator of the HA-dependent function of ITIH5, we found a significant impact of ITIH5 on risk for recurrence only in bladder cancers with strong CD44 expression. Given the proposed ITIH5-HA-C44 axis as main mechanism of cancer cell suppression, our data provide a rational why ITIH5 might be predominantly down-regulated in basal-type cancers, i.e., in those tumors known to be characterized by abundant CD44 expression. Only in this setting, the secreted ECM-modulator ITIH5 may gain its full tumor suppressive potential reflected by longer relapse-free survival of bladder cancer patients while its down-regulation gives tumors advantages to grow and metastasize. ITIH5 and CD44 protein data supported this hypothesis although only a tendency of an inverse expression was found while, again, associated with a putative impact of ITIH5 on patients’ RFS in tumors with strong CD44 expression.

In vitro, we confirmed a tumor suppressive role of ITIH5 in CD44 expressing SCaBER cells reflecting the BASQ subtype but not in basal-type HT1376 cancer cells with lower CD44 (variant) expression. Consistent with previous reports [20,21,22], ITIH5 expression impaired tumor cell and colony growth as well as cell capacities to colonize an artificial wound in vitro. ITIH5 expression further enhanced adhesion of SCaBER cells on HA, supporting involvement of the ITIH5-HA-CD44 axis in cell adhesion. This observation is in line with previous studies for ITIH5 [22] and other members of the ITI heavy chain family [41]. Transcriptomic profiling of ITIH5 expressing SCaBER cells showed enrichment of genes involved in ECM remodeling and signaling transduction. In 2017, we firstly presented data proposing involvement of ITIH5 in ECM remodeling, affecting the ECM-cell interaction in MDA-MB-231 breast cancer cells [22]. These cells exhibited altered organization of focal adhesion (FA) points associated with increased cell contractility and a shift to a low motile and low invasive phenotype of breast tumor cells [22]. These previous findings supported our notion that ITIH5 modulates mechanotransductive downstream signaling affecting ECM-cell interactions and finally, migration behavior of breast cancer cells.

Interestingly, in SCaBER cells, we now observed comparable effects of ITIH5 re-expression on FA reorganization, resulting in reduction of FA size and number. This new finding emphasizes the fundamental role of ITIH5 as direct or indirect modulator of matrix adhesion-mediated motility of BASQ cells as well. This was validated by a substantial reduction of cell migration in our wound healing assays, indicating a low invasive bladder cell phenotype after ITIH5 re-expression. In line with the here observed reduced FA size in ITIH5 clones, other work demonstrated that FA size predicts migration speed of highly invasive fibrosacroma cells [42]. However, so far, for FA numbers, no such clear correlation has been shown in bladder cancer.

Beyond that, Huth and colleagues previously revealed impaired development of keratinocytes in a 3D skin model due to ITIH5 loss [17] which was recently added by genome-wide expression data of ITIH5 knockout mice [16]. Based on that involvement of ITIH5 in biological processes like wound healing, epidermis development or ECM organization has been verified [16]. Bearing in mind that skin studies also confirmed HA stabilization by ITIH5, a general and tissue independent impact of ITIH5 on ECM remodeling and phenotype differentiation of epithelial cells could be assumed. Of clinical significance, we provide functional evidence that ITIH5 expression may support response of bladder cancers to chemotherapeutics. In 2010, a gene pattern including ITIH5 has been identified, potentially modulating the sensitivity for anti-microtubule drug-mediated chemotherapy [43]. Apart from that, the impact of ITIH5 on anti-cancer treatment remained elusive although CD44 and distinct splice variants have been intensively studied, highlighting an impact on cellular responsiveness to chemotherapeutics [44]. Hagiwara and colleagues observed, for instance, involvement of CD44 v8–10 in acquired resistance of urothelial cancer cells to cisplatin [35]. Bourguignon summarized that the HA-CD44-mediated signaling regulates, for instance, non-coding RNAs such as the long-coding RNAs UCA1, which contribute to chemoresistance [45]. In addition, ECM components such as collagens including COL12A1 are thought to affect sensitivity of cancer cells to chemotherapeutics [34,46]. In line with that, ITIH5 expression associated with altered ECM-cell interaction significantly enhanced sensitivity of SCaBER to both cisplatin and gemcitabine up to 15-fold. Still, the European Association of Urology recommends a cisplatin-based chemotherapy as first line therapy of muscle-invasive and metastatic bladder cancer which is usually combined with gemcitabine [47]. Thus, ITIH5 might be of interest to develop novel panels of predictive biomarkers helping to stratify bladder cancer patients for (neo)adjuvant treatment. ITIH5 DNA methylation, which was shown to be useful as biomarker to detect bladder cancer via urine [24], may further hold information for predicting chemo-response of bladder cancer patients.

In conclusion, we gained insight into the tumor suppressive role of ITIH5 in (basal-type) bladder cancer demonstrating an impact on standard therapy approaches in vitro. Underlying mechanisms may involve the HA-CD44 axis, however, the role of ITIH5 independently of HA or CD44 is not fully understood and required further investigations especially according to a putative inverse regulation with CD44. Knowledge about the function of the protein domains, i.e., vault protein inter-alpha-trypsin (VIT) and von Willebrand type A (vWA), which are conserved in all heavy chains [48], is poor. As both, i.e., our TCGA-based survival curve analyses and the in vitro findings based on HT1376 cells, indicate contrary (prognostic) effects of ITIH5, further studies addressing ITIH5 mechanisms are required which should also consider the role of different splice variants of the CD44 receptor.

## 4. Materials and Methods

### 4.1. Reagents and Cell Lines

The chemotherapeutics, gemcitabine and cisplatin, were obtained ready-to-use from the in-house pharmacy of the RWTH Aachen University Hospital. The urothelial cancer cell line HT1376 (basal-type) was obtained from the American Type Culture Collection (ATCC, Manassas, VA, USA). SCaBER, a basal-type bladder cancer cell line with squamous characteristics, was a kind gift of Prof. Wolfgang Schulz/Dr. Michèle Hoffmann (Düsseldorf University Hospital, Düsseldorf, Germany). All cell lines were cultured using the DMEM (Dulbecco’s Modified Eagle’s Medium) (Sigma-Aldrich, Deisenhofen, Germany) supplemented with 10% FCS (Gibco Laboratories, Berlin, Germany), and successfully underwent an identity check (Multiplexion GmbH, Immenstadt, Germany) prior to the experiments. All cells and clones were regularly tested for mycoplasma infection using the PCR-based Venor^®^ GeM Mycoplasma Detection Kit (Minerva Biolabs, Berlin, Germany).

### 4.2. Clinical Patient Samples

Bladder cancers were retrospectively collected from pathology archives in Aachen (*n* = 52). For cohort characteristics, see Table 2. Tissue microarrays of formalin-fixed paraffin-embedded (FFPE) surgical specimens were used as previously described [49,50,51]. The RWTH University Hospital Aachen Local Ethics Committee approved the retrospective, pseudonymized study of archival tissues (RWTH EK 172/16).

### 4.3. Stable Transfection of SCaBER and HT1376 Single-Cell Clones

Transfection of SCaBER and HT1376 wildtype cells with either the ITIH5-pBK-CMV expression vector, containing the full-length human ITIH5 cDNA, or the empty vector alone was performed as recently specified [22]. Single-cell clones were selected and cultured by limited dilution under geneticin (G418; Thermo Fisher Scientific, Waltham, MA, USA) pressure.

### 4.4. Nucleic Acid Extraction and Reverse Transcription PCR

Total RNA from cultured cells was prepared by using TRIzol reagent (Invitrogen Life Technologies, Darmstadt, Germany). cDNA synthetization was performed using the reverse transcription system (Promega, Madison, WI, USA) according to the manufacturer’s instructions.

### 4.5. Real-Time qPCR

ITIH5 mRNA expression was analyzed by semi-quantitative real-time PCR using SYBR-Green PCR mix (Bio-Rad Laboratories, Munich, Germany) and performed in an iCycler IQ5 (Bio-Rad Laboratories). Gene expression was quantified by using the comparative ΔΔ-C_T_ method calculating relative expression values [52]. The reference gene GAPDH was used as reference gene. For primers used in this study, see our previous publication [22].

### 4.6. Western Blot

Western blot analysis was performed to assess ITIH5 expression as well as integrin (β-integrins 1, 3, 4 and α5-integrin) protein levels as recently described [22].

### 4.7. Immunofluorescence and FA Quantification

SCaBER cells, cultivated for 24 h on fibronectin (FN) coated glass surfaces, were fixed and stained as reported previously [53]. Cells were permeabilized with 1% Triton X100 (Sigma-Aldrich, St. Louis, MO, USA) in cytoskeleton-buffer (CB). Primary antibody (anti-vinculin clone hVin-1, V19131 Sigma-Aldrich) were incubated overnight and secondary antibodies coupled with fluorescent dyes (546 donkey anti-mouse, A10036) as well as Alexa Atto 633 (1862, all Sigma-Aldrich) for actin staining were applied for 45 min. Nuclei were counterstained with NucBlue (R37606, ThermoFisher, Waltham, MA, USA). Samples were imaged using a confocal laser scanning microscope 880 (LSM880 with Ayryscan detector, Zeiss, Germany) with a Zeiss EC-Plan-Neofluar oil immersion objective lens (40×, NA = 1.4) and Zeiss software (ZEN 2.3).

For FA quantification, Imaris 9.1 software (Bitplane, Zürich, Switzerland) was used to detect the fluorescence signal for vinculin (patch number and size) in individual samples. Imaris-surface detection was performed according to the following parameters: Manual Threshold Value: 6.06628; Diameter of largest sphere: 0.8 µm; Surface Filter-lower Threshold Manual Value: 143 voxel.

### 4.8. Immunohistochemistry

ITIH5 immunohistochemical staining was performed on TMA FFPE sections with the DAKO 5001 Kit (DAKO, Hamburg, Germany) and incubated with the anti-ITIH5 antibody (Pineda Company, Berlin, Germany) as previously described [20]. For CD44 staining, the monoclonal anti-CD44 (clone DF1485 (Agilent Technologies, Inc., Santa Clara, CA, USA), PTlink pH 6, dilution 1:200, Flex+M; DAKO) was used. After incubation with primary antibody, DAKO EnVision^TM^FLEX system (mouse linker and horseradish peroxidase-conjugated polymer) for detection was applied. Reactions were visualized with DAKO Liquid DAB Substrate Chromogen System and hematoxylin counterstain. Stainings were evaluated by an experienced uropathologist (N.T.G.). Staining intensities (0 = no staining, 1 = weak staining, 2 = moderate staining, 3 = strong staining) and percentages of positive-stained viable tumor cells according to the system of Remmele and Stegner (IRS) were reported [54].

### 4.9. Cell Growth Assay

Stable SCaBER and HT-1376 clones were grown in triplicates in a 6-well plate (1 × 10^4^ cells/well). At distinct time points, i.e., 24, 48, 72 and 96 h after cell seeding, cell number of viable cells was determined by using the automated CASY-1 cell counter system (Schärfe System, Reutlingen, Germany).

### 4.10. Cell Attachment Assay

Cell-matrix adhesion on hyaluronan (100 µg/mL; Sigma-Aldrich) was performed as reported [22].

### 4.11. Colony Formation Assay

Analysis of colony forming and growth was performed in 6-well plates, culturing the cells over 10 days as previously described [20].

### 4.12. Wound Healing Assay

Capacities of cells to colonize a scratched area were studied by using an in vitro monolayer wound healing assay as specified [20].

### 4.13. Drug Response Analyses

Dose response analyses were performed using the XTT Cell viability assay (Roche Diagnostics, Penzberg, Germany) according to the manufacturer’s instructions. SCaBER cells (mock and ITIH5 clones) were treated with various doses of cisplatin (0.01–100 µM in H_2_O), and gemcitabine (0.0001–1000 µM in H_2_O). Logarithmic transformation, normalization (defining smallest value = 0% and largest value = 100%) and non-linear regression of raw data was performed using GraphPad Prism 6 software (GraphPad Software Inc., La Jolla, CA, USA). The relative inhibition rate (100% − X^inh^) and the IC_50_ (drug concentration causing 50% inhibition) values for each cell line and mock/ITIH5 clone were determined using “log (inhibitor) vs. normalized response–variable slope” equation considering the extra sum-of-squares F Test to prove significance of the null hypothesis (IC_50_ same or all data sets). If *p* value is <0.05, so the null hypothesis that curves have the same IC_50_ is rejected.

### 4.14. TCGA BLCA Data Set Analyses

Public BLCA data sets from the Cancer Genome Atlas (TCGA) [55] network including RNASeqV2 data (level 3) of tumor and normal tissue samples were analyzed and sub-classified as previously described [31]. In order to assess associations between ITIH5/CD44 mRNA expression and patients’ outcome, both follow-up and clinico-pathological data were obtained using cBioPortal (https://www.cbioportal.org/, accessed on 23 April 2021, [56,57]) filtering for the BLCA patient IDs.

### 4.15. Gene Expression Profiling

Total RNA samples were processed with Affymetrix GeneChipTM WT Plus Reagent Kit (Affymetrix, CA, USA) according to standard protocol. The resulted end-labeled cDNAs were hybridized on the GeneChipTM HTA 2.0 Human arrays (Affymetrix, CA, USA) for 16 h, at 60 rpm, at 45 °C in a Hybridization Oven 645 (Affymetrix, CA, USA). Arrays were scanned on an Affymetrix 3000 7G scanner (Affymetrix, CA, USA), after washing and staining on an Affymetrix 450 Fluidics Station (Affymetrix, CA, USA) as described in the manufacturer’s manual. CEL files were generated in GeneChip Command Console Software (Affymetrix, CA, USA) with default settings after the scan. Gene expression intensities were normalized and summarized with robust multiarray average algorithm (RMA) [58], which was implemented in program package of AltAnalyze (version 2.1.3) [59]. Differential expression analysis were also performed with AltAnalyze packages. Genes with a fold change greater than 1.25 and a raw *p*-value (moderate *t*-test) less than 0.01 were identified as up- or down-regulated genes.

The microarray data were uploaded to the National Center for Biotechnology Information Gene Expression Omnibus (GSE167320). Overlap of significantly down- or up-regulated genes with Molecular Signature Data Base (MSigDB) gene sets between ΔpBK-SC-ITIH5 and ΔpBK-SC-mock clones was performed using a public gene set enrichment analysis (GSEA) platform (GSEA; http://www.broadinstitute.org/gsea/index.jsp, accessed on 23 April 2021) [34].

### 4.16. Statistical Data Acquisition

Two-sided *p*-values less than 0.05 were considered significant. In order to compare two groups, the non-parametric Mann–Whitney U-test was used. Correlation analysis was performed by calculating a non-parametric Spearman’s rank correlation coefficient. Survival curves for recurrence-free survival (RFS) were calculated using the Kaplan–Meier method with log-rank statistics using SPSS software version 22.0 (SPSS Inc., Chicago, IL, USA). RFS was measured from surgery until relapse (local/distant) and was censored for patients without evidence of tumor recurrence at the last follow-up date.

## Figures and Tables

**Table 1:** Annotated biological processes (BP), biological functions (BF) and cellular components (CC) associated with ITIH5.

GO Category	GO Ontology	GO Term	*n* Changed	*n* Measured	*n* in Gene-Set	*p*-Value	*z*-Score
GO:0032862	BP	activation of Rho GTPase activity	3	22	23	0.004	4.662
GO:0005576	BP	extracellular region	41	1535	1803	<0.001	3.891
GO:0016049	BP	cell growth	4	91	100	0.049	2.259
GO:0004871	BF	signal transducer activity	34	1545	1911	0.028	2.315
GO:0005576	CC	extracellular region	56	2061	2438	<0.001	4.761

**Table 2:** Clinico-pathological parameters of 52 bladder cancer specimens immunohistochemically analyzed in this study.

Parameter	Categorization	n	% Analyzable
Age at diagnosis (median: 72)	<70 years	26	50.0
	≥70 years	26	50.0
Gender			
	female	7	13.5
	male	45	86.5
Histological tumor grade			
	low grade	0	0.00
	high grade	52	100
Tumor stage			
	pTa	14	26.9
	pT1	28	53.8
	pT2	5	9.6
	pT3	4	7.7
	pT4	1	1.9

Only patients with primary bladder cancers without any neoadjuvant therapy were included.

## Data Availability

The data that support the findings of this study are available from the corresponding author upon reasonable request.

## Supplementary Materials

The following are available online at https://www.mdpi.com/article/10.3390/cells10051038/s1, Supplementary Table S1: List of ITIH5-associated genes significantly up- and down-regulated in SCaBER clones, Supplementary Table S2: Results of GSEA analyses.
