# Supplementary material for: The ECM Modulator ITIH5 Affects Cell Adhesion, Motility and Chemotherapeutic Response of Basal/Squamous-Like (BASQ) Bladder Cancer Cells
Source: Cells. 2021 Apr 28;10(5):1038. doi: 10.3390/cells10051038 (PMC8146567; doi:10.3390/cells10051038)
Supplement: Supplementary file 1 [file cells-10-01038-s001.zip › cells-1167245-supplementary/Supplementary Table 2.pdf]

Supplementary Table 2: Overlap of ITIH5-regulated genes in SCaBER with published gene sets (GSEA)

Gene Set Name (GO)	# Genes in Gene Set (K)	# Genes in Overlap (k)	k/K	p-value	FDR q-value
MOLECULAR_TRANSDUCER_ACTIVITY	1479	35	0.0237	1.03E-9	1.36E-5
SIGNALING_RECEPTOR_BINDING	1654	35	0.0212	1.76E-8	5.79E-5
REGULATION_OF_CELL_DIFFERENTIATION	1945	38	0.0195	3.33E-8	7.31E-5
COLLAGEN_CONTAINING_EXTRACELLULAR_MATRIX	427	16	0.0375	1.21E-7	1.59E-4
EXTRACELLULAR_MATRIX	568	18	0.0317	2.36E-7	2.59E-4
